# Periodontitis induced by *Porphyromonas gingivalis* drives impaired glucose metabolism in mice

**DOI:** 10.3389/fcimb.2022.998600

**Published:** 2022-10-10

**Authors:** Ni Kang, Yong Zhang, Fei Xue, Jinyu Duan, Fan Chen, Yu Cai, Qingxian Luan

**Affiliations:** ^1^ Department of Periodontology, Peking University School and Hospital of Stomatology and National Center of Stomatology and National Clinical Research Center for Oral Diseases and National Engineering Research Center of Oral Biomaterials and Digital Medical Devices and Beijing Key Laboratory of Digital Stomatology and Research Center of Engineering and Technology for Computerized Dentistry Ministry of Health and National Medical Products Administration (NMPA) Key Laboratory for Dental Materials, Beijing, China; ^2^ Central Laboratory, Peking University School and Hospital of Stomatology and National Center of Stomatology and National Clinical Research Center for Oral Diseases and National Engineering Research Center of Oral Biomaterials and Digital Medical Devices and Beijing Key Laboratory of Digital Stomatology and Research Center of Engineering and Technology for Computerized Dentistry Ministry of Health and National Medical Products Administration (NMPA) Key Laboratory for Dental Materials, Beijing, China; ^3^ First Clinical Division, Peking University School and Hospital of Stomatology and National Center of Stomatology and National Clinical Research Center for Oral Diseases and National Engineering Research Center of Oral Biomaterials and Digital Medical Devices and Beijing Key Laboratory of Digital Stomatology and Research Center of Engineering and Technology for Computerized Dentistry Ministry of Health and National Medical Products Administration (NMPA) Key Laboratory for Dental Materials, Beijing, China; ^4^ Department of Stomatology, People’s Hospital of Peking University, Beijing, China

**Keywords:** periodontitis, *Porphyromonas gingivalis* (*P. gingivalis*), glucose metabolism, inflammation, alveolar bone loss

## Abstract

Periodontitis has been demonstrated to be bidirectionally associated with diabetes and has been recognized as a complication of diabetes. As a periodontal pathogen, *Porphyromonas gingivalis* is a possible pathogen linking periodontal disease and systemic diseases. It has also been found to be involved in the occurrence and development of diabetes. In this study, 6-week-old male C57BL/6 mice were orally administered the *P. gingivalis* strain ATCC381 for 22 weeks. Histological analysis of the gingival tissue and quantified analysis of alveolar bone loss were performed to evaluate periodontal destruction. Body weight, fasting glucose, glucose tolerance test (GTT), and insulin tolerance test (ITT) were used to evaluate glucose metabolism disorder. We then analyzed the expression profiles of inflammatory cytokines and chemokines in gingival tissue, the liver, and adipose tissue, as well as in serum. The results showed that mice in the *P. gingivalis*-administered group developed apparent gingival inflammation and more alveolar bone loss compared to the control group. After 22 weeks of *P. gingivalis* infection, significant differences were observed at 30 and 60 min for the GTT and at 15 min for the ITT. *P. gingivalis*-administered mice showed an increase in the mRNA expression levels of the pro-inflammatory cytokines (TNF-α, IL-6, IL-17, and IL-23) and chemokines (CCL2, CCL8, and CXCL10) in the gingiva and serum. The expression levels of the glucose metabolism-related genes were also changed in the liver and adipose tissue. Our results indicate that oral administration of *P. gingivalis* can induce changes in the inflammatory cytokines and chemokines in the gingiva and blood, can lead to alveolar bone loss and to inflammatory changes in the liver and adipose tissues, and can promote glucose metabolism disorder in mice.

## Introduction

Periodontitis is a chronic infectious disease caused by a dental plaque microbiome. It features connective tissue destruction and alveolar bone loss. A series of studies have demonstrated that periodontitis not only causes tooth loss but also contributes to the progress of systemic diseases; it has also been recognized as a complication of diabetes (Cullinan and Seymour, 2013). It has been established that diabetes has bidirectional positive associations with periodontitis and that high prevalence and severe periodontitis in diabetes mellitus patients may influence glycemic control (Nascimento et al., 2018; Sthr et al., 2021). The underlying mechanisms linking periodontitis and diabetes have been considered to be pro-inflammatory cytokines derived from gingival lesions [tumor necrosis factor alpha (TNF-α), interleukin 6 (IL-6), and IL-17]. These are chronically overexpressed in advanced periodontitis and can be released into the blood to affect other organs and exacerbate metabolic diseases (Kashiwagi et al., 2021); however, several reports have shown that the levels of serum TNF-α and other pro-inflammatory cytokines in periodontitis patients are not elevated compared with those of healthy subjects (Yamazaki et al., 2005). Thus, the pathogenic mechanisms of periodontitis affecting diabetes remain to be elucidated.

Recent studies have shown that *Porphyromonas gingivalis*, a major pathogenic bacterium in periodontitis, is a possible pathogen linking periodontal disease to other systemic diseases (Darveau et al., 2012). It has been found to participate in the occurrence and development of insulin resistance (Makiura et al., 2008; Aemaimanan et al., 2013; Arimatsu et al., 2014; Bhat et al., 2014). The levels of HbA1c have been shown to be related to the number of red complex bacteria, including *P. gingivalis*, in periodontal tissues (Aemaimanan et al., 2013). *P. gingivalis* infection increased systemic inflammation, especially in adipose tissue, through the induction of IL-6, which can induce insulin resistance (Pradhan et al., 2001). Oral administration of *P. gingivalis* has also been observed to elicit endotoxemia and induce insulin resistance in mice (Arimatsu et al., 2014).

To demonstrate the causal role of periodontal disease as a risk factor for diabetes, we orally administered *P. gingivalis* to mice. Alveolar bone loss levels were evaluated and glucose metabolism was measured. Inflammatory mediators and the expression levels of glucose metabolism-related genes were also investigated. We report the observation of a causal role for *P. gingivalis*, a periodontitis-related pathogen, in the progress of diabetes and metabolic disorders. We have shown that oral administration of *P. gingivalis* has an impact on serum inflammatory cytokines, chemokines, and the expression levels of glucose-related genes in liver and adipose tissue, resulting in metabolic disorders. Our results also confirm that *P. gingivalis* can aggravate impaired glucose metabolism. This may enhance our knowledge of the mechanistic relationship between periodontitis and the clinical features of diabetes.

## Materials and methods

### Ethics statement

All animal experimental procedures in this study were approved by the Animal Welfare Ethics of Peking University Biomedical Ethics Committee (LA2018142).

### Mice

Twelve 4-week-old C57BL/6 male mice, weighing nearly 20 g (Vital River Inc., Beijing, China) were purchased and group-housed (six mice per cage) in a specific pathogen-free environment. The mice were fed regular chow and sterile water until the commencement of infection at 6 weeks of age. During the observation period, body weight and food intake were monitored every other week.

### Bacterial cultures

The *P. gingivalis* strain ATCC381 was cultured on anaerobic basal agar plates (Oxoid, Oxoid Ltd., Hampshire, England) enriched with 5% sheep blood and incubated under anaerobic conditions (80% N_2_, 10% CO_2_, and 10% H_2_) for 3–5 days at 37°C. Cultures were then inoculated into brain heart infusion broth (Oxoid, Oxoid Ltd., Hampshire, England), supplemented with 5 μg/ml hemin and 0.4 μg/ml menadione (Sigma-Aldrich, St. Louis, MO, USA), and grown for 2 days based on a calibration curve of optical density measured at 600 nm *versus* the viable cell count in colony forming units (CFU) per milliliter, corresponding to 10^9^ CFU/ml. The cultured cells were then centrifuged at 8,000 × *g* for 20 min at 4°C and suspended in phosphate-buffered saline (PBS) with 2% carboxymethylcellulose (CMC) (Sigma-Aldrich, St. Louis, MO, USA) for oral administration to increase viscosity.

### Induction of diabetes and periodontitis in mice

Twelve mice were randomly divided into two groups: the control group and the *P.gingivalis*-administered group. *P. gingivalis* and CMC were administered orally through a plastic tube, with 10^8^ CFU *P. gingivalis* in 100 μl PBS mixed with 2% CMC (for the *P. gingivalis*-administered group) or only 100 μl PBS mixed with 2% CMC (for the control group) every 2 days for 22 weeks. The mice were then sacrificed after anesthetization. The blood, gingiva, liver, adipose tissue, and maxilla samples were excised and harvested for the following experiments.

### Tissue collection and preparation

Blood samples were collected *via* infraorbital puncture. Serum was isolated by centrifugation at 10,000 rpm for 5 min at 4°C. The gingiva, liver, and the epididymal white adipose tissues were collected from each mouse and were rapidly removed from the mice, placed into a liquid nitrogen box, and kept at −80°C until analyzed, or fixed by 10% formalin for histological staining. Mouse maxillae were harvested and fixed in 4% paraformaldehyde at 4°C overnight, and then transferred to a 70% ethanol solution. Horizontal bone loss around the maxillary molars was examined using micro-CT.

### Glucose and insulin tolerance tests

Glucose tolerance test (GTT) and insulin tolerance test (ITT) were performed. For the GTT, the fasting glucose levels were measured following overnight (12 h) fasting. Thereafter, the mice were injected intraperitoneally with 1.5 g of glucose (Solarbio, Beijing, China) per kilogram of body weight. Blood samples were collected through the tail tip vein at 30, 60, and 90 min after injection. For the ITT, following 4 h of fasting, the mice were injected intraperitoneally with 2 U of insulin (Actrapid MC, Novo Nordisk, Bagsværd, Denmark) per kilogram of body weight. Blood samples were collected through the tail vein at 15, 30, 45, and 60 min after injection. The glucose levels were determined using a glucometer (Sinocare, Hunan, China).

### Quantification of maxillary alveolar bone resorption

Micro-CT imaging of the mouse hemi-maxillae, free of soft tissues, was performed using a CT scanner (Inveon MM Gantry-STD 3121, Siemens, Munich, Germany) for the purpose of generating three-dimensional models. The parameters were as follows: 360° rotation, 360 projections, 1,500-ms exposure time, 60-kV source voltage, 220-μA beam current, 8.82-μm effective pixel size, and 18-μm isotropic resolution. Acquisitions were reconstructed with a filtered back-projection algorithm, matrix size of 1,024 × 1,024 × 448, using Inveon Acquisition Workplace software (COBRA, Siemens, Berlin, Germany). The images were rotated and adjusted from M1 to M3 and then analyzed by an examiner (NK) blinded to the experimental groups. Inveon Research Workplace software (COBRA, Siemens, Berlin, Germany) was used to measure the distance from the cemento-enamel junction (CEJ) to the alveolar bone crest (ABC) at six points: 1) mesiobuccal and 2) distobuccal regions of the first maxillary molar; 3) mesiobuccal and 4) distobuccal regions of the second maxillary molar; and 5) mesiobuccal and 6) distobuccal regions of the third maxillary molar. Measurements were taken for the purpose of evaluating the levels of alveolar bone loss (ABL). Prior to the observation, the intraclass correlation for the evaluation of bone loss measurements was calculated. The same examiner evaluated the same tooth points on different days, and the intraclass correction coefficient (ICC) was 0.89.

### Serum cytokine, chemokine, and receptor levels

Serum samples were isolated from the blood after euthanasia. Commercially available ELISA kits were used to determine the serum concentrations of cytokines (TNF-α, IL-6, IL-17, and IL-23) (RayBiotech Inc., Norcross, GA, USA), chemokines and receptor chemokines [C–C motif ligand 2 (CCL2), C–C motif receptor 2 (CCR2), CCL3, CCL8, C–X–C motif ligand 10 (CXCL10), and CCL20] (Mei Mian, Jiangsu, China), and developmental endothelial locus-1 (Del-1) (Signalway Antibody Co., Ltd., Pearland, TX, USA) according to the manufacturers’ protocols.

### RNA isolation and quantitative real-time PCR analysis

Total RNAs from the gingiva, liver, and adipose tissue were extracted using an RNeasy Mini kit (Qiagen, Valencia, CA, USA) according to the manufacturer’s instructions. Total RNA was quantified by measuring the absorbance at 260–280 nm. Subsequently, aliquots of RNA were reverse transcribed to complementary DNA (cDNA) using a Primescript RT Master Mix Kit (Takara Bio, Shiga, Japan). Quantitative real-time PCR analysis was performed using an Applied Biosystems 7500 Fast Real-time PCR System (Life Technology, Carlsbad, CA, USA) in accordance with the manufacturer’s protocol. Briefly, the reactions were carried in a 10-μl mixture of 2× SYBR Green (Takara Bio, Shiga, Japan), each primed at 100 nM and 30 ng of reverse-transcribed RNA. The PCR program consisted of a 30-s incubation at 95°C, followed by 40 cycles at 95°C for 5 s and 60°C for 34 s. A dissociation curve analysis was then performed to confirm specificity. Each gene was tested in triplicate. The relative level of messenger RNA (mRNA) for each target mRNA was determined using the 2^−ΔΔ^ *^C^* ^t^ method. The relative quantities were normalized to glyceraldehyde 3-phosphate dehydrogenase (*GAPDH*) mRNA and represented in fold changes relative to those from control group mice. The sequences of the primers are provided in **Table 1**.

**Table 1:** Primer sequences used for real-time PCR.

Gene	Forward	Reverse
*GAPDH*	AGGTTGTCTCCTGCGACTTCA	CTGTTGCTGTAGCCGTATTCATTG
*TNF-α*	AGGCGGTGCCTATGTCTCAG	GCCATTTGGGAACTTCTCATC
*IL-6*	TAGCTACCTGGAGTACATGAAGAACA	TGGTCCTTAGCCACTCCTTCTG
*IL-17*	CCTCAGACTACCTCAACCGTTC	ACTGAGCTTCCCAGATCACAGAG
*IL-23*	GAGCAACTTCACACCTCCCTACTA	TGCCACTGCTGACTAGAACTCA
*Del-1*	CTTGGTAGCAGCCTGGCTTT	GCCTTCTGGACACTCACAGG
*CCL2*	GCATCCACGTGTTGGCTCA	CTCCAGCCTACTCATTGGGATCA
*CCL8*	CGCAGTGCTTCTTTGCCTG	TCTGGCCCAGTCAGCTTCTC
*CXCL10*	AGAACGGTGCGCTGCAC	CCTATGGCCCTGGGTCTCA
*CCR2*	ATCCACGGCATACTATCAACATC	CAAGGCTCACCATCATCGTAG
*CCL20*	TTTTGGGATGGAATTGGACAC	TGCAGGTGAAGCCTTCAACC
*CCL3*	CCACTGCCCTTGCTGTTCTT	GCAAAGGCTGCTGG-TTTCAA
*G6pc*	CAGCAAGGTAGATCCGGGA	AAAAAGCCAACGTATGGATTCCG
*Saa*	GTAATTGGGGTCTTTGCC	TTCTGCTCCCTGCTCCTG
*Pck1*	AAGTGCCTGCACTCTGTGG	CAGGCCCAGTTGTTGACC
*Pparα*	GTCATCACAGACACCCTC	TATTCGACACTCGATGTTCAG
*Irs1*	CTTCTCAGACGTGCGCAAGG	GTTGATGTTGAAACAGCTCTC
*Fitm2*	ATGGAGCACCTGGAGCGC	TCATTTCTTGTAAGTATCTCGCTTCAAAG
*Angptl4*	CCAACGCCACCCACTTAC	CTCGGTTCCCTGTGATGC
*C1qtnf9*	CCTGCACACCAAGGACAGTTAC	TGTCACCTGCATCCACACTTC
*Sirt1*	TTCACATTGCATGTGTGTGG	TAGCCTGCGTAGTGTTGGTG

### Statistical analysis

All results are presented as the mean ± standard deviation (SD). Data between two groups were analyzed using unpaired two-tailed Student’s *t*-test. Multiple comparisons were analyzed with one-way analysis of variance (ANOVA) followed by Tukey–Kramer multiple tests using SPSS 20.0 statistical software (SPSS Inc., IBM, Chicago, IL, USA) and GraphPad Prism v8.0.2 (GraphPad Software, San Diego, CA, USA). *P* < 0.05 was considered to indicate statistical significance.

## Results

### *P. gingivalis* administration had no significant effect on animal body weight

**Figure 1:**
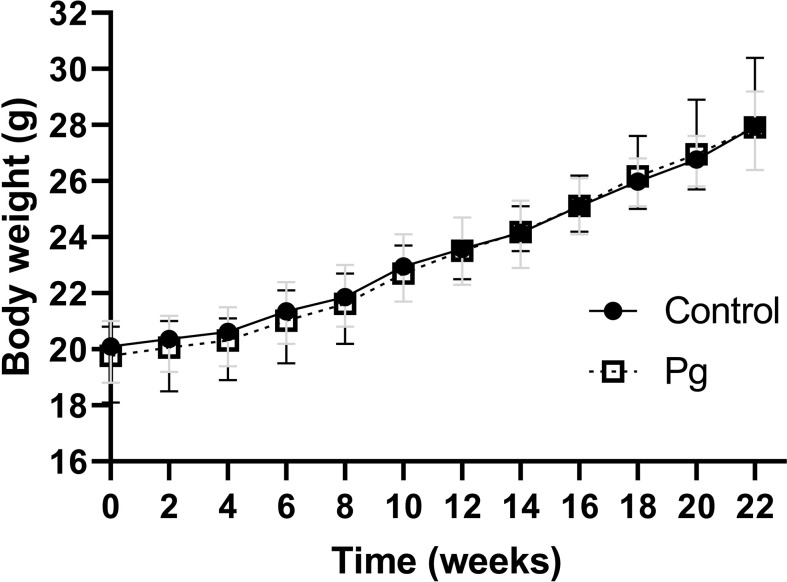
Effects of the oral administration of *Porphyromonas gingivalis* on body weight. Body changes during the experimental period in the control group (*N* = 6) and the *P. gingivalis*-administered group (*N* = 6) were measured. All data for each time point are expressed as the mean ± SEM.

There was no significant difference in body weight from the baseline of the experiment after treatment with *P. gingivalis* for 22 weeks. In addition, there was no significant difference in body weight or food intake between the *P. gingivalis*-administered group and the control group (**Figure 1**).

### *P. gingivalis* Administration induced alveolar bone loss

**Figure 2:**
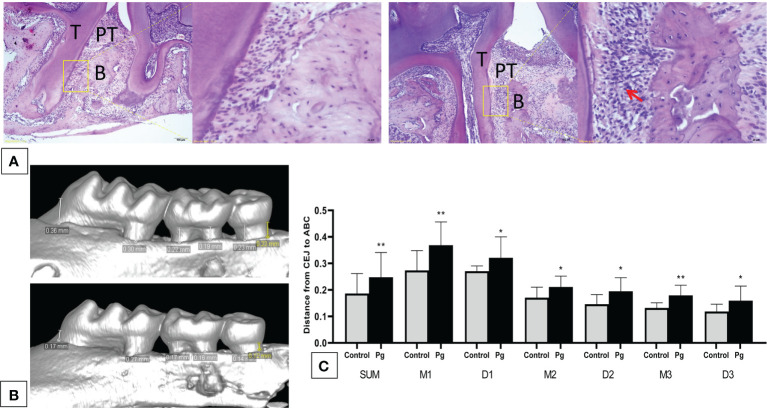
Oral administration of *Porphyromonas gingivalis* for 22 weeks induced periodontitis. **(A)** Histological examination of the hemi-maxillae (*T*, tooth; *PT*, periodontal tissue; *B*, alveolar bone) stained with hematoxylin–eosin (H&E). Apparent losses of periodontal attachment and alveolar bone resorption. The *arrow* shows inflammatory cells that have infiltrated the periodontal tissue. **(B)** Buccal side maxillary alveolar bone loss measured from the cemento-enamel junction (CEJ) to the alveolar bone crest (ABC) at six points—*1*) mesiobuccal and *2*) distobuccal regions for the first maxillary molar; *3*) mesiobuccal and *4*) distobuccal regions for the second maxillary molar; and *5*) mesiobuccal and *6*) distobuccal regions for the third maxillary molar—in mice from the *P. gingivalis-*administered group and the control group. **(C)** comparison of the six CEJ–ABC linear distances (*SUM*, the sum of six points; *M1*, mesiobuccal for the first maxillary molar; *D1*, distobuccal for the first maxillary molar; *M2*, mesiobuccal for the second maxillary molar; *D2*, distobuccal for the second maxillary molar; *M3*, mesiobuccal for the third maxillary molar; *D3*, distobuccal for the third maxillary molar). *p < 0.05, **p < 0.01. *N* = 6. Data are expressed as the mean ± SEM.

We showed *via* hematoxylin–eosin (H&E) staining that inflammatory cells infiltrated the periodontal tissue of mice (**Figure 2A**), and apparent gingival inflammation was observed after oral *P. gingivalis* infection. Mice in the *P. gingivalis*-administered group exhibited significantly more alveolar bone loss compared to those in the control group (**Figure 2B**). Quantification of bone resorption indicated a statistical increase at all six points in the *P. gingivalis*-administered group compared to the control group (the sum of the six points; 0.18 ± 0.07 mm *vs*. 0.25 ± 0.09 mm for the control and *P. gingivalis*, respectively, *p* < 0.01) (**Figure 2C**). Based on these data, we conclude that repeated oral administration of *P. gingivalis* can induce alveolar bone resorption.

### *P. gingivalis* administration induced an inflammatory response in gingival tissue

**Figure 3:**
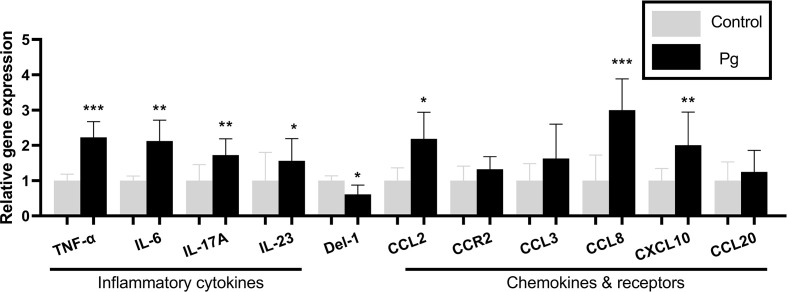
Effects of the oral administration of *Porphyromonas gingivalis* on the gene expressions of inflammatory cytokines and chemokines in the gingiva of mice in the *P. gingivalis*-administered group and the control group. The relative mRNA expressions of the genes of interest were normalized to the relative quantity of glyceraldehyde 3-phosphate dehydrogenase (*GAPDH*) mRNA. *p < 0.05, **p < 0.01, ***p < 0.001. *N* = 6. Data are expressed as the mean ± SEM.

To examine the inflammatory mediators in periodontitis induced by *P. gingivalis*, the expression levels of the genes in the gingiva from the two groups of mice were determined using real-time PCR. The *P. gingivalis*-administered group showed an increase in the mRNA expression levels of the pro-inflammatory cytokines (TNF-α, IL-6, IL-17, and IL-23; *p* < 0.05) and chemokines (CCL2, CCL8 and CXCL10; *p* < 0.05). We also observed a decrease in the mRNA expression levels of the anti-inflammatory mediator (Del-1; *p* < 0.05). However, there was no significant difference in the gingival gene expressions of CCR2, CCL3, and CCL20 between the two groups (**Figure 3**).

### *P. gingivalis* administration induced impaired glucose metabolism

**Figure 4:**
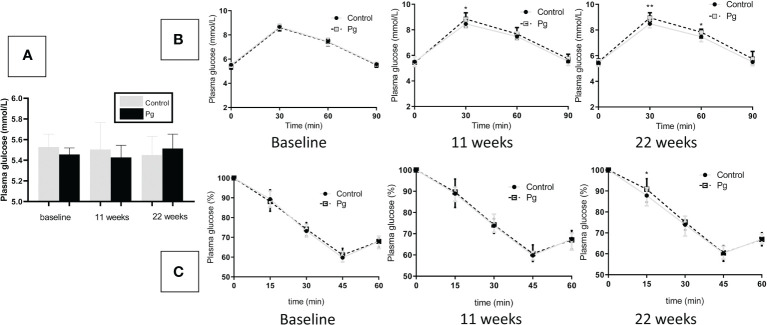
Effects of the oral administration of *Porphyromonas gingivalis* on fasting glucose, glucose tolerance, and insulin sensitivity. **(A)** Fasting glucose levels between the *P. gingivalis*-administered group and the control group at baseline, 11 weeks, and at 22 weeks after *P. gingivalis* infection. **(B)** Plasma glucose levels during an intraperitoneal glucose tolerance test (GTT) following overnight (12 h) fasting at baseline, 11 weeks, and at 22 weeks after *P. gingivalis* infection. **(C)** Plasma glucose levels during an intraperitoneal insulin tolerance test (ITT) following 4 h of fasting at baseline, 11 weeks, and at 22 weeks after *P. gingivalis* infection. *p < 0.05, **p < 0.01. *N* = 6. Data are expressed as the mean ± SEM.

To explore the effect of oral administration of *P. gingivalis* on the glucose metabolism of mice, we performed fasting GTT and ITT at baseline, 11 weeks, and at 22 weeks of oral *P. gingivalis* administration. As shown in **Figure 4A**, at baseline, there were no significant differences in the fasting glucose levels and the GTT and ITT results between the control group and the experimental group. At 11 and 22 weeks after *P. gingivalis* infection, the fasting glucose levels showed no significant difference between these two groups. Although the effect of *P. gingivalis* infection was relatively weak, at 11 weeks after *P. gingivalis* administration, a significant difference was observed at 30 min for the GTT. At 22 weeks after *P. gingivalis* infection, in the *P. gingivalis*-administered group, significant differences were observed at 30 and 60 min for the GTT and at 15 min for the ITT. An overall effect was evident, as shown in **Figures 4B, C**.

### *P. gingivalis* administration induced an inflammation- and glucose metabolism-related gene response in the liver

**Figure 5:**
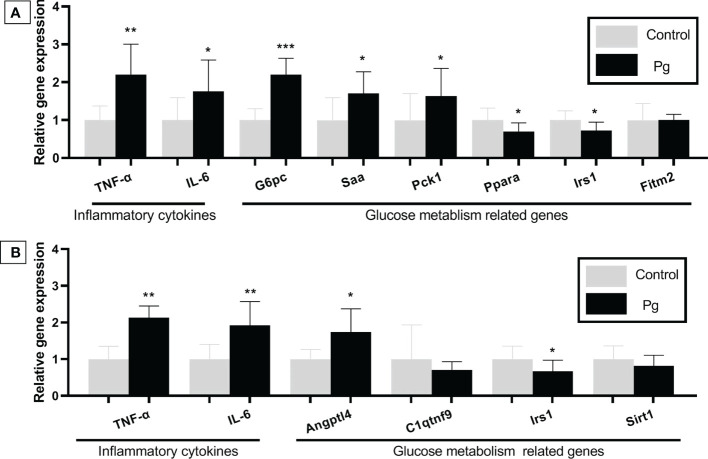
Effects of the oral administration of *Porphyromonas gingivalis* on the gene expression of inflammatory cytokines and the expression of the glucose metabolism-related genes in the liver **(A)** and adipose tissue **(B)** of mice in the *P. gingivalis*-administered group and the control group. The relative mRNA expressions of the genes of interest were normalized to the relative quantity of glyceraldehyde 3-phosphate dehydrogenase (*GAPDH*) mRNA. *p < 0.05, **p < 0.01, ***p < 0.001. *N* = 6. Data are expressed as the mean ± SEM.

We compared the gene expression profiles of live tissue samples from mice in the *P. gingivalis*-administered group and the control group. Oral administration of *P. gingivalis* led to increased mRNA expressions of the pro-inflammatory cytokines TNF-α and IL-6 and the expression levels of genes related to glucose metabolism [negative regulation effect genes: glucose 6 phosphatase (*G6pc*), serum amyloid A (*Saa*), and phosphoenolpyruvate carboxykinase 1 (*Pck1*)]. The mRNA expressions of peroxisome proliferator activated receptor alpha (*Ppara*) and insulin receptor substrate (*Irs1*), which positively regulate glucose metabolism, were downregulated in the *P. gingivalis*-administered group (**Figure 5A**). There was no significant difference in the expression of fat storage-inducing transmembrane protein 2 (*Fitm2*) between these two groups.

### *P. gingivalis* administration induced an inflammation- and glucose metabolism-related gene response in adipose tissue

TNF-α and IL-6 are known to be pro-inflammatory and are adipocytokines that are expressed in adipocytes to modulate glucose metabolism by suppressing insulin signals (Pradhan et al., 2001). The mRNA expressions of *TNF-α*, *IL-6*, and angiopoietin-like 4 (*Angptl4*), which are involved in insulin resistance (Watanabe et al., 2008; Trayhurn and Alomar, 2015), were shown to be higher in the adipose tissue of mice from the *P. gingivalis*-administered group. Conversely, the *Irs1* gene, which improves insulin sensitivity (Copps and White, 2012), was downregulated in the *P. gingivalis*-administered group. There was no significant difference in the expressions of c1q/tnf-related protein 9 (*C1qtnf*9) and sirtuin type 1 (*Sirt*1) between the two groups (**Figure 5B**).

### *P. gingivalis* administration increased serum cytokine and chemokine levels

**Figure 6:**
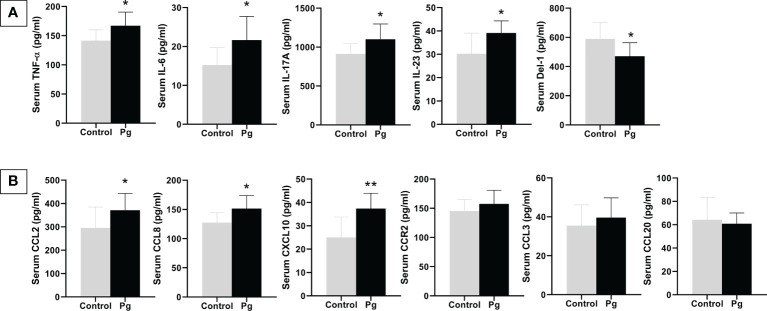
Effects of the oral administration of *Porphyromonas gingivalis* on serum inflammatory cytokines **(A)** and chemokines **(B)** between mice in the *P. gingivalis*-administered group and the control group determined by ELISA. *p < 0.05, **p < 0.01. *N* = 6. Data are expressed as the mean ± SEM.

We compared the levels of inflammatory cytokines and chemokines in serum between the *P. gingivalis*-administered group and the control group. As shown in **Figure 6**, *P. gingivalis* infection increased the serum levels of the pro-inflammatory cytokines TNF-α, IL-6, IL-17A, and IL-23 and the chemokines CCL2, CCL8, and CXCL10. The serum level of the potential anti-inflammatory mediator Del-1 was decreased in the *P. gingivalis*-administered group compared to the control group. Similar to the results of the gingival mRNA gene expression levels, there was no significant difference in serum CCR2, CCL3, and CCL20 between the two groups.

## Discussion

Periodontitis is believed to be bidirectionally related to diabetes. Clinical investigations have confirmed that periodontitis increases the severity of diabetes (Kuo et al., 2008; Genco et al., 2020; Genco and Borgnakke, 2020) and that periodontal treatment can improve glycemic control in diabetes patients (Lalla et al., 2006; D'Aiuto et al., 2018). However, the mutually promotional relationship between periodontitis and diabetes is still not fully understood.

Numerous studies have explored the role of periodontitis in the incidence of diabetes in animal investigations and have shown that oral administration of periodontal bacteria diminished glucose tolerance and insulin sensitivity in mice (Arimatsu et al., 2014; Komazaki et al., 2017). In this study, after oral *P. gingivalis* infection, the levels of fasting glucose showed no significant difference compared to those in the control group. However, after GTT, mice in the *P. gingivalis* group showed high blood glucose levels 30 min after glucose injection at 11 weeks after *P. gingivalis* infection. Furthermore, the mice in this group displayed high blood glucose 30 and 60 min after glucose injection at 22 weeks after *P. gingivalis* infection. After ITT, the mice in the *P. gingivalis* group showed high blood glucose 15 min after insulin injection at 22 weeks after *P. gingivalis* infection. Although these effects were relatively modest, we confirmed that mice could develop impaired glucose metabolism after repeated oral administration of *P. gingivalis* (Sasaki et al., 2018; Seyama et al., 2020; Tian et al., 2020). However, some researchers have shown inconsistent or contradictory results. The study by Mahamed et al. (2005) showed that periodontitis is not associated with the onset and severity of diabetes after oral administration of *P. gingivalis*. The studies of Li et al. (2013); Wang et al. (2016); Ahn et al. (2021), and Ohtsu et al. (2019) showed no significant difference in glucose metabolism between *P. gingivalis*-infected and sham-infected mice. These discrepancies might be due to differences in the procedures of oral infection and the measures of glucose metabolism. Our study administered *P. gingivalis* infection every 2 days for 22 weeks, Li et al. carried out *P. gingivalis* infection in three batches during the experimental period and three times at 2-day intervals per batch, and Ahn and Ohtsu administered *P. gingivalis* infection only for 6 or 5 weeks and calculated the fasting glucose levels without performing GTT or ITT.

In previous studies, TNF-α and IL-6 had been proven to be pro-inflammatory cytokines in periodontal tissues (Kurgan and Kantarci, 2018). Moreover, they are also recognized as adipocytokines with fairly negative impacts on insulin signaling (Pradhan et al., 2001); the combination of TNF-α and IL-6 had an impact on progressive pancreatic β-cell loss during the progression of diabetes (Watanabe et al., 2008; Ilievski et al., 2017). In this study, we compared the gingival and serum levels of TNF-α and IL-6 between the *P. gingivalis* administration and control groups and found that the gingival mRNA expression levels of *TNF-α* and *IL-6* were upregulated after *P. gingivalis* infection and that their serum levels were also significantly increased. These results reveal that oral *P. gingivalis* infection induced an increase in gingival and serum TNF-α and IL-6. This had harmful effects on insulin sensitivity and was attributable to the progression of impaired glucose metabolism.

Low-grade inflammation is considered the common systemic effect of periodontitis (Preshaw et al., 2012; Hajishengallis and Chavakis, 2021b). IL-17 and IL-23 are pro-inflammatory cytokines that can cause dysbiosis in bacterial communities and are associated with leukocyte adhesion (Abusleme and Moutsopoulos, 2017). IL-17 and Del-1 have exhibited an antagonistic relationship in inflammatory effects (Saxena et al., 2020; Hajishengallis and Chavakis, 2021a). In addition, they have been observed to play a critical role in the loss of pancreatic B cells and have been shown to have pro-diabetic effects (Mensah-Brown et al., 2006; Marwaha et al., 2014). In the present study, we observed an upregulated mRNA expression of *IL-17* and a downregulated mRNA expression of *Del-1* in the gingiva. Similar changes were observed in the serum levels of IL-17 and IL-23, which were increased, and the potential anti-inflammatory mediator Del-1, which was decreased in the *P. gingivalis* group compared to the control group.

Chemokines have also been observed to be involved in the pathogenesis of insulin resistance and diabetes (Esser et al., 2014). IL-17 can increase the expression and the production of CCL2 and CXCL10 to enhance its pro-inflammatory effect (Matsui and Yoshida, 2016). CCL2 (Elmarakby and Sullivan, 2012; Ota, 2013), CCL8 (Sarkar et al., 2012), and CXCL10 (Antonelli et al., 2014; Hueso et al., 2018; Moreno et al., 2022) have also been demonstrated to play a role in the development of diabetes. Along with the elevation of IL-17, the gingival mRNA expressions of *CCL2*, *CCL8*, and *CXCL10* were upregulated. Similar alterations were seen in serum, CCL2, CCL8, and CXCL10 were increased in the *P. gingivalis* group compared to the control group. All of these results provide evidence that elevated chemokine levels induced by IL-17 after *P. gingivalis* infection may be related to the progression of glucose metabolism disorder.

The liver is crucial for maintaining normal glucose homeostasis and regulating glycogen synthesis and degradation, glycolysis, and gluconeogenesis, depending on the fasting and postprandial states. Accompanied by changes of the cytokines and chemokines in the gingiva and serum, the expressions of *TNF-α* and *IL-6* were upregulated in the livers of mice in the *P. gingivalis* group. In addition, the expression levels of the glucose metabolism-related genes *G6pc* (Bruni et al., 1999; Pound et al., 2013), *Saa* (Leinonen et al., 2003; Han et al., 2007; Sjöholm et al., 2009), and *Pck*1 (Choi et al., 2008; Yuan et al., 2019), which can influence gluconeogenesis, were upregulated in the livers of mice in the *P. gingivalis* group. The expression levels of the genes *Ppara* (Srinivasan et al., 2004; Maciejewska-Skrendo et al., 2019) and *Irs1* (Dong et al., 2006; Copps and White, 2012; Takatani et al., 2021), which have been reported to improve insulin sensitivity, were downregulated in the livers of *P. gingivalis*-administered mice. Adipose tissue also plays an important role in mediating glucose metabolism and insulin sensitivity through various molecules, including the adipocytokines TNF-α and IL-6, which possess strong, naturally pro-inflammatory traits that negatively impact insulin signaling. In the present study, the expression levels of *TNF-α* and *IL-6* were significantly upregulated in the adipose tissue from the *P. gingivalis*-administered group. Additionally, an upregulated expression of the pro-inflammatory gene *Angptl*4 (Trayhurn and Alomar, 2015), which is supposed to increase insulin resistance, was observed; on the other hand, *Irs1* (Copps and White, 2012), a gene that improves insulin sensitivity, was downregulated. Taken together, these results indicate that the oral administration of *P. gingivalis* has harmful effects on the inflammatory response and on insulin sensitivity. Although the effect was not robust, continuous deterioration of these cytokines and chemokines could have significant negative effects on both glucose tolerance and insulin sensitivity. The above results confirm that the proposed mechanism for periodontitis-induced diabetes is the increase of the expressions of the inflammatory cytokines and chemokines produced in periodontal lesions.

One limitation of our study is that the symptoms of impaired glucose metabolism were mild. Therefore, further investigations using an increased dosage of bacterial infection, shorter intervals of bacterial incubation, or combined treatment with molar ligation might be targeted for future study.

In the present study, we demonstrated that the oral administration of *P. gingivalis* induced alveolar bone loss and inflammatory changes in the liver and adipose tissues, which also induced impaired glucose metabolism after GTT and ITT. These changes were considered to be attributable to the changes in the levels of the inflammatory cytokines (TNF-α, IL-6, IL-17, and IL-23), chemokines (CCL2, CCL8, and CXCL10), and another mediator (Del-1) in the gingiva and blood. They provide further insight into the role of *P. gingivalis*-mediated inflammatory cytokines and chemokines in the development of diabetes. However, it remains to be elucidated whether a causal relationship exists between periodontitis and impaired glucose metabolism. Further investigations are needed to examine whether other oral periodontal pathogens have similar effects on glucose metabolism.

## Data availability statement

The original contributions presented in the study are included in the article/supplementary material. Further inquiries can be directed to the corresponding authors.

## Ethics statement

This study was reviewed and approved by the Animal Welfare Ethics of Peking University Biomedical Ethics Committee.

## Author contributions

NK and YZ contributed equally to the conception, experimentation, and preparation of the manuscript and figures. FX, JD, and FC participated in the experimentation and preparation of the manuscript, including text and figures. YC and QL provided supervision and wrote and edited the manuscript. All authors contributed to the article and approved the submitted version.

## Funding

This study was supported by the National Natural Science Foundation of China (project no. 81800978). The funder had no role in the design of the study, collection, analysis or interpretation of the data, or in writing the manuscript.

## Conflict of interest

The authors declare that the research was conducted in the absence of any commercial or financial relationships that could be construed as a potential conflict of interest.

## Publisher’s note

All claims expressed in this article are solely those of the authors and do not necessarily represent those of their affiliated organizations, or those of the publisher, the editors and the reviewers. Any product that may be evaluated in this article, or claim that may be made by its manufacturer, is not guaranteed or endorsed by the publisher.

## References

[B1] Abusleme L. Moutsopoulos N. M. (2017). IL-17: overview and role in oral immunity and microbiome. Oral. Dis. 23, 854–865. doi: 10.1111/odi.12598 27763707 PMC5398954

[B2] Aemaimanan P. Amimanan P. Taweechaisupapong S. (2013). Quantification of key periodontal pathogens in insulin-dependent type 2 diabetic and non-diabetic patients with generalized chronic periodontitis. Anaerobe 22, 64–68. doi: 10.1016/j.anaerobe.2013.06.010 23827459

[B3] Ahn J. S. Yang J. W. Oh S. J. Shin Y. Y. Kang M. J. Park H. R.. (2021). Porphyromonas gingivalis exacerbates the progression of fatty liver disease *via* CD36-PPARgamma pathway. BMB Rep. 54, 323–328. doi: 10.5483/BMBRep.2021.54.6.050 34078528 PMC8249874

[B4] Antonelli A. Ferrari S. M. Corrado A. Ferrannini E. Fallahi P. (2014). CXCR3, CXCL10 and type 1 diabetes. Cytokine Growth Factor Rev. 25, 57–65. doi: 10.1016/j.cytogfr.2014.01.006 24529741

[B5] Arimatsu K. Yamada H. Miyazawa H. Minagawa T. Nakajima M. Ryder M. I.. (2014). Oral pathobiont induces systemic inflammation and metabolic changes associated with alteration of gut microbiota. Sci. Rep. 4, 4828. doi: 10.1038/srep04828 24797416 PMC4010932

[B6] Bhat U. G. Ilievski V. Unterman T. G. Watanabe K. (2014). Porphyromonas gingivalis lipopolysaccharide upregulates insulin secretion from pancreatic beta cell line MIN6. J. Periodontol. 85, 1629–1636. doi: 10.1902/jop.2014.140070 24921432 PMC4394373

[B7] Bruni N. Rajas F. Montano S. Chevalier-Porst F. Mithieux G. (1999). Enzymatic characterization of four new mutations in the glucose-6 phosphatase (G6PC) gene which cause glycogen storage disease type 1a. Ann. Hum. Genet. 63, 141–146. doi: 10.1046/j.1469-1809.1999.6320141.x 10738525

[B8] Choi M. S. Jung U. J. Yeo J. Kim M. J. Lee M. K. (2008). Genistein and daidzein prevent diabetes onset by elevating insulin level and altering hepatic gluconeogenic and lipogenic enzyme activities in non-obese diabetic (NOD) mice. Diabetes Metab. Res. Rev. 24, 74–81. doi: 10.1002/dmrr.780 17932873

[B9] Copps K. D. White M. F. (2012). Regulation of insulin sensitivity by serine/threonine phosphorylation of insulin receptor substrate proteins IRS1 and IRS2. Diabetologia 55, 2565–2582. doi: 10.1007/s00125-012-2644-8 22869320 PMC4011499

[B10] Cullinan M. P. Seymour G. J. (2013). Periodontal disease and systemic illness: will the evidence ever be enough? Periodontol. 2000 62, 271–286. doi: 10.1111/prd.12007 23574472

[B11] D'Aiuto F. Gkranias N. Bhowruth D. Khan T. Orlandi M. Suvan J.. (2018). Systemic effects of periodontitis treatment in patients with type 2 diabetes: a 12 month, single-centre, investigator-masked, randomised trial. Lancet Diabetes Endocrinol. 6, 954–965. doi: 10.1016/S2213-8587(18)30038-X 30472992

[B12] Darveau R. P. Hajishengallis G. Curtis M. A. (2012). Porphyromonas gingivalis as a potential community activist for disease. J. Dent. Res. 91, 816–820. doi: 10.1177/0022034512453589 22772362 PMC3420389

[B13] Dong X. Park S. Lin X. Copps K. White M. F. (2006). Irs1 and Irs2 signaling is essential for glucose homeostastis and systemic growth. J. Clin. Invest. 116, 101–114. doi: 10.1172/JCI25735 16374520 PMC1319221

[B14] Elmarakby A. A. Sullivan J. C. (2012). Relationship between oxidative stress and inflammatory cytokines in diabetic nephropathy. Cardiovasc. Ther. 30, 49–59. doi: 10.1111/j.1755-5922.2010.00218.x 20718759

[B15] Esser N. Legrand-Poels S. Piette J. Scheen A. J. Paquot N. (2014). Inflammation as a link between obesity, metabolic syndrome and type 2 diabetes. Diabetes Res. Clin. Pract. 105, 141–150. doi: 10.1016/j.diabres.2014.04.006 24798950

[B16] Genco R. J. Borgnakke W. S. (2020). Diabetes as a potential risk for periodontitis: association studies. Periodontol. 2000 83, 40–45. doi: 10.1111/prd.12270 32385881

[B17] Genco R. J. Graziani F. Hasturk H. (2020). Effects of periodontal disease on glycemic control, complications, and incidence of diabetes mellitus. Periodontol. 2000 83, 59–65. doi: 10.1111/prd.12271 32385875

[B18] Hajishengallis G. Chavakis T. (2021a). DEL-1: a potential therapeutic target in inflammatory and autoimmune disease? Expert Rev. Clin. Immunol. 17, 549–552. doi: 10.1080/1744666X.2021.1915771 33870840 PMC8405457

[B19] Hajishengallis G. Chavakis T. (2021b). Local and systemic mechanisms linking periodontal disease and inflammatory comorbidities. Nat. Rev. Immunol. 21, 426–440. doi: 10.1038/s41577-020-00488-6 33510490 PMC7841384

[B20] Han C. Y. Subramanian S. Chan C. K. Omer M. Chiba T. Wight T. N.. (2007). Adipocyte-derived serum amyloid A3 and hyaluronan play a role in monocyte recruitment and adhesion. Diabetes 56, 2260–2273. doi: 10.2337/db07-0218 17563062

[B21] Hueso L. Ortega R. Selles F. Wu-Xiong N. Y. Ortega J. Civera M.. (2018). Upregulation of angiostatic chemokines IP-10/CXCL10 and I-TAC/CXCL11 in human obesity and their implication for adipose tissue angiogenesis. Int. J. Obes. 42, 1406–1417. doi: 10.1038/s41366-018-0102-5 29795466

[B22] Ilievski V. Bhat U. G. Suleiman-Ata S. Bauer B. A. Toth P. T. Olson S. T.. (2017). Oral application of a periodontal pathogen impacts SerpinE1 expression and pancreatic islet architecture in prediabetes. J. Periodontal Res. 52, 1032–1041. doi: 10.1111/jre.12474 28643938 PMC5668151

[B23] Kashiwagi Y. Aburaya S. Sugiyama N. Narukawa Y. Sakamoto Y. Takahashi M.. (2021). Porphyromonas gingivalis induces entero-hepatic metabolic derangements with alteration of gut microbiota in a type 2 diabetes mouse model. Sci. Rep. 11, 18398. doi: 10.1038/s41598-021-97868-2 34526589 PMC8443650

[B24] Komazaki R. Katagiri S. Takahashi H. Maekawa S. Shiba T. Takeuchi Y.. (2017). Periodontal pathogenic bacteria, aggregatibacter actinomycetemcomitans affect non-alcoholic fatty liver disease by altering gut microbiota and glucose metabolism. Sci. Rep. 7, 13950. doi: 10.1038/s41598-017-14260-9 29066788 PMC5655179

[B25] Kuo L. C. Polson A. M. Kang T. (2008). Associations between periodontal diseases and systemic diseases: a review of the inter-relationships and interactions with diabetes, respiratory diseases, cardiovascular diseases and osteoporosis. Public Health 122, 417–433. doi: 10.1016/j.puhe.2007.07.004 18028967

[B26] Kurgan S. Kantarci A. (2018). Molecular basis for immunohistochemical and inflammatory changes during progression of gingivitis to periodontitis. Periodontol. 2000 76, 51–67. doi: 10.1111/prd.12146 29194785

[B27] Lalla E. Cheng B. Lal S. Tucker S. Greenberg E. Goland R.. (2006). Periodontal changes in children and adolescents with diabetes: a case-control study. Diabetes Care 29, 295–299. doi: 10.2337/diacare.29.02.06.dc05-1355 16443876

[B28] Leinonen E. Hurt-Camejo E. Wiklund O. Hultén L. M. Hiukka A. Taskinen M. R. (2003). Insulin resistance and adiposity correlate with acute-phase reaction and soluble cell adhesion molecules in type 2 diabetes. Atherosclerosis 166, 387–394. doi: 10.1016/s0021-9150(02)00371-4 12535753

[B29] Li H. Yang H. Ding Y. Aprecio R. Zhang W. Wang Q.. (2013). Experimental periodontitis induced by porphyromonas gingivalis does not alter the onset or severity of diabetes in mice. J. Periodontal Res. 48, 582–590. doi: 10.1111/jre.12041 23317150

[B30] Maciejewska-Skrendo A. Buryta M. Czarny W. Król P. Spieszny M. Stastny P.. (2019). The polymorphisms of the peroxisome-proliferator activated receptors' Alfa gene modify the aerobic training induced changes of cholesterol and glucose. J. Clin. Med. 8, 1043. doi: 10.3390/jcm8071043 PMC6679124 31319591

[B31] Mahamed D. A. Marleau A. Alnaeeli M. Singh B. Zhang X. Penninger J. M.. (2005). G(-) anaerobes-reactive CD4+ T-cells trigger RANKL-mediated enhanced alveolar bone loss in diabetic NOD mice. Diabetes 54, 1477–1486. doi: 10.2337/diabetes.54.5.1477 15855336

[B32] Makiura N. Ojima M. Kou Y. Furuta N. Okahashi N. Shizukuishi S.. (2008). Relationship of porphyromonas gingivalis with glycemic level in patients with type 2 diabetes following periodontal treatment. Oral. Microbiol. Immunol. 23, 348–351. doi: 10.1111/j.1399-302X.2007.00426.x 18582336

[B33] Marwaha A. K. Tan S. Dutz J. P. (2014). Targeting the IL-17/IFN-gamma axis as a potential new clinical therapy for type 1 diabetes. Clin. Immunol. 154, 84–89. doi: 10.1016/j.clim.2014.06.006 24947953

[B34] Matsui T. Yoshida Y. (2016). Reduction of the expression and production of adhesion molecules and chemokines by brain endothelial cells in response to tumor necrosis factor-α and interleukin-17 in hypothermia. Clin. Exp. Neuroimmunol. 7, 174–182. doi: 10.1111/cen3.12298

[B35] Mensah-Brown E. P. Shahin A. Al-Shamisi M. Wei X. Lukic M. L. (2006). IL-23 leads to diabetes induction after subdiabetogenic treatment with multiple low doses of streptozotocin. Eur. J. Immunol. 36, 216–223. doi: 10.1002/eji.200535325 16358360

[B36] Moreno B. Hueso L. Ortega R. Benito E. Martínez-Hervas S. Peiro M.. (2022). Association of chemokines IP-10/CXCL10 and I-TAC/CXCL11 with insulin resistance and enhance leukocyte endothelial arrest in obesity. Microvascular Res. 139, 1042–1054. doi: 10.1016/j.mvr.2021.104254 34534571

[B37] Nascimento G. G. Leite Fábio R.M. Vestergaard P. Scheutz F. López R. (2018). Does diabetes increase the risk of periodontitis? a systematic review and meta-regression analysis of longitudinal prospective studies. Acta Diabetologica 55, 653–667. doi: 10.1016/j.mvr.2021.104254 29502214

[B38] Ohtsu A. Takeuchi Y. Katagiri S. Suda W. Maekawa S. Shiba T.. (2019). Influence of porphyromonas gingivalis in gut microbiota of streptozotocin-induced diabetic mice. Oral. Dis. 25, 868–880. doi: 10.1111/odi.13044 30667148

[B39] Ota T. (2013). Chemokine systems link obesity to insulin resistance. Diabetes Metab. J. 37, 165–172. doi: 10.4093/dmj.2013.37.3.165 23807918 PMC3689012

[B40] Pound L. D. Oeser J. K. O'Brien T. P. Wang Y. Faulman C. J. Dadi P. K.. (2013). G6PC2: a negative regulator of basal glucose-stimulated insulin secretion. Diabetes 62, 1547–1556. doi: 10.2337/db12-1067 23274894 PMC3636628

[B41] Pradhan A. D. Manson J. E. Rifai N. Buring J. E. Ridker P. M. (2001). C-reactive protein, interleukin 6, and risk of developing type 2 diabetes mellitus. JAMA 286, 327–334. doi: 10.1001/jama.286.3.327 11466099

[B42] Preshaw P. M. Alba A. L. Herrera D. Jepsen S. Konstantinidis A. Makrilakis K.. (2012). Periodontitis and diabetes: a two-way relationship. Diabetologia 55, 21–31. doi: 10.1007/s00125-011-2342-y 22057194 PMC3228943

[B43] Sarkar S. A. Lee C. E. Victorino F. Nguyen T. T. Walters J. A. Burrack A. (2012) Expression and regulation of chemokines in murine and human type 1 diabetes. Diabetes 61, 436–446. doi: 10.2337/db11-0853 22210319 PMC3266427

[B44] Sasaki N. Katagiri S. Komazaki R. Watanabe K. Maekawa S. Shiba T.. (2018). Endotoxemia by porphyromonas gingivalis injection aggravates non-alcoholic fatty liver disease, disrupts Glucose/Lipid metabolism, and alters gut microbiota in mice. Front. Microbiol. 9. doi: 10.3389/fmicb.2018.02470 PMC6207869 30405551

[B45] Saxena S. Venugopal R. Chandrayan Rao R. Yuwanati M. B. Awasthi H. Jain M. (2020). Association of chronic periodontitis and type 2 diabetes mellitus with salivary del-1 and IL-17 levels. J. Oral. Biol. Craniofac Res. 10, 529–534. doi: 10.1016/j.jobcr.2020.08.013 32874883 PMC7452335

[B46] Seyama M. Yoshida K. Yoshida K. Fujiwara N. Ono K. Eguchi T.. (2020). Outer membrane vesicles of porphyromonas gingivalis attenuate insulin sensitivity by delivering gingipains to the liver. Biochim. Biophys. Acta Mol. Basis Dis. 1866, 165731. doi: 10.1016/j.bbadis.2020.165731 32088316

[B47] Sjöholm K. Lundgren M. Olsson M. Eriksson J. W. (2009) Association of serum amyloid a levels with adipocyte size and serum levels of adipokines: differences between men and women Cytokine 48, 260–266. doi: 10.1016/j.cyto.2009.08.005 19758820

[B48] Srinivasan S. Hatley M. E. Reilly K. B. Danziger E. C. Hedrick C. C. (2004). Modulation of PPARalpha expression and inflammatory interleukin-6 production by chronic glucose increases monocyte/endothelial adhesion. Arterioscler. Thromb. Vasc. Biol. 24, 851–857. doi: 10.1161/01.ATV.zhq0504.2260 15001458

[B49] Sthr J. Barbaresko J. Neuenschwander M. Schlesinger S. (2021). Bidirectional association between periodontal disease and diabetes mellitus: a systematic review and meta-analysis of cohort studies. Sci. Rep. 11, 13686. doi: 10.1038/s41598-021-93062-6 34211029 PMC8249442

[B50] Takatani T. Shirakawa J. Shibue K. Gupta M. K. Kulkarni R. N. (2021). Insulin receptor substrate 1 (IRS1), but not IRS2, plays a dominant role in regulating pancreatic alpha cell function in mice. J. Biol. Chem. 296, 100646. doi: 10.1016/j.jbc.2021.100646 33839150 PMC8131928

[B51] Tian J. Liu C. Zheng X. Jia X. Peng X. Yang R.. (2020). Porphyromonas gingivalis induces insulin resistance by increasing BCAA levels in mice. J. Dent. Res. 99, 839–846. doi: 10.1177/0022034520911037 32176550

[B52] Trayhurn P. Alomar S. Y. (2015). Oxygen deprivation and the cellular response to hypoxia in adipocytes - perspectives on white and brown adipose tissues in obesity. Front. Endocrinol. (Lausanne) 6. doi: 10.3389/fendo.2015.00019 PMC4333869 25745415

[B53] Wang Q. Zhang P. Aprecio R. Zhang D. Li H. Ji N.. (2016). Comparison of experimental diabetic periodontitis induced by porphyromonas gingivalis in mice. J. Diabetes Res. 2016, 4840203. doi: 10.1155/2016/4840203 27995146 PMC5141310

[B54] Watanabe K. Petro B. J. Shlimon A. E. Unterman T. G. (2008). Effect of periodontitis on insulin resistance and the onset of type 2 diabetes mellitus in zucker diabetic fatty rats. J. Periodontol. 79, 1208–1216. doi: 10.1902/jop.2008.070605 18597603

[B55] Yamazaki K. Honda T. Oda T. Ueki-Maruyama K. Nakajima T. Yoshie H. Seymour G. J. (2005). Effect of periodontal treatment on the c-reactive protein and proinflammatory cytokine levels in Japanese periodontitis patients. J. Periodontal Res. 40, 53–58. doi: 10.1111/j.1600-0765.2004.00772.x 15613080

[B56] Yuan X. Dong D. Li Z. Wu B. (2019). Rev-erbα activation down-regulates hepatic Pck1 enzyme to lower plasma glucose in mice. Pharmacol. Res. 141, 310–318. doi: 10.1016/j.phrs.2019.01.010 30639375

